# The Benefits of Prone SPECT Myocardial Perfusion Imaging in Reducing Both Artifact Defects and Patient Radiation Exposure

**DOI:** 10.5935/abc.20150122

**Published:** 2015-10

**Authors:** Maria Stathaki, Sophia Koukouraki, Emmanouela Papadaki, Angeliki Tsaroucha, Nikolaos Karkavitsas

**Affiliations:** Department of Nuclear Medicine, University Hospital of Heraklion, Crete – Greece

**Keywords:** Prone Position, Myocardial Perfusion, Radioactive Emission, Technetium (Tc-99m) Tetrofosmin, SPECT instead of Tomography emission-computed single-photon

## Abstract

**Background:**

Prone imaging has been demonstrated to minimize diaphragmatic and breast tissue attenuation.

**Objectives:**

To determine the role of prone imaging on the reduction of unnecessary rest perfusion studies and coronary angiographies performed, thus decreasing investigation time and radiation exposure.

**Methods:**

We examined 139 patients, 120 with an inferior wall and 19 with an anterior wall perfusion defect that might represented attenuation artifact. Post-stress images were acquired in both the supine and prone position. Coronary angiography was used as the “gold standard” for evaluating coronary artery patency. The study was terminated and rest imaging was obviated in the presence of complete improvement of the defect in the prone position. Quantitative interpretation was performed. Results were compared with clinical data and coronary angiographic findings.

**Results:**

Prone acquisition correctly revealed defect improvement in 89 patients (89/120) with inferior wall and 12 patients (12/19) with anterior wall attenuation artifact. Quantitative analysis demonstrated statistically significant difference in the mean summed stress scores (SSS) of supine and mean SSS of prone studies in patients with disappearing inferior wall defect in the prone position and patent right coronary artery (true negative results). The mean difference between SSS in supine and in prone position was higher with disappearing than with remaining defects.

**Conclusion:**

Technetium-99m (Tc-99m) tetrofosmin myocardial perfusion imaging with the patient in the prone position overcomes soft tissue attenuation; moreover it provides an inexpensive, accurate approach to limit the number of unnecessary rest perfusion studies and coronary angiographies performed.

## Introduction

Myocardial perfusion imaging has become an effective clinical tool for diagnosing coronary artery disease (CAD), risk stratifying of patients after infarction, assessing myocardial viability and planning therapy^1,2^ and is usually performed with the patient in the supine position^3,4^. It is, however, recognized that the diaphragmatic attenuation of the inferior wall and the breast attenuation of the anterior wall in females, has an impact on the test specificity^1,3-5^. Planar acquisition, prone imaging, ECG gating and image quantitation constitute commonly used approaches to overcome soft tissue attenuation. Although direct approaches for attenuation correction have been commercially available, they are quite expensive and possibly not provided to all nuclear medicine departments^1,6^.

Prone imaging has been reported to improve inferior wall attenuation artifact by producing an anterior shifting of the heart and lowering of the diaphragm and subdiaphragmatic organs^4,5^. Normal prone scans in patients with inferior wall defects in the supine images are associated with low cardiac event rates, similar to that of patients with normal supine‑only studies^4,7,8^. The main pitfall of this imaging approach is that sternal and rib attenuation may create an anterior or anteroseptal wall defect^1,4^. In addition, the technique seems to be less suitable for reducing attenuation from the breast tissue^9,10^.

Although stress studies have traditionally been followed by several hour-rest delayed images, the normal stress-only approach is recently preferred^8^, as it is less time-consuming, reduces radiation exposure and has an excellent short-term prognosis^4,6,8^. In the presence of an inferior wall perfusion defect in the stress-supine study, positional change (prone imaging) is a low cost, effective and clinically validated technique to overcome diaphragmatic attenuation artifacts^5,7^.

The purpose of this study was initially to confirm the impact of the supine and prone approaches on attenuation artifacts. Additionally, we investigated its role in reducing subsequent rest imaging and unnecessary referrals to coronary arteriography, aiming to decrease investigation and hospital waiting time, patient discomfort and also radiation exposure.

## Methods

### Study population

We examined 139 patients, 120 with an inferior wall and 19 with an anterior wall perfusion defect. The clinical characteristics of the patients are shown in Table 1. Post‑stress images were acquired in both the supine and prone position. Coronary angiography was used as the “gold standard” for identifying coronary vessels patency. In many instances, scintigraphy was performed within 6 months of coronary angiography so as to evaluate the success of revascularization and/or to determine the hemodynamic significance of coronary stenosis, the adequacy of collateral circulation and the risk stratification of known CAD. In some cases myocardial perfusion imaging was followed by coronary angiography, in order to determine coronary artery narrowing of a scintigraphically-demonstrated ischemia and/or to evaluate patients with inexplicable chest pain. In all cases, the time interval between coronary angiography and scintigraphy was limited to no more than 6 months. The aforementioned criteria defined the size of our sample. The clinical indications for myocardial perfusion imaging are shown in Table 2. There was no case of dominant left circumflex artery (LCx), which could also be relevant to inferior wall defects.

**Table 1:** Patients' Characteristics

Parameter			Value
Number of patients			139
Age (years)			65.8 ± 11.6
Sex (male : female)			114 (82%):25 (18%)
**Perfusion defect location**			
		Inferior wall	120 (86.4%)
		Anterior wall	19 (13.6%)
Hypertension			72 (51.7%)
Diabetes			33 (23.7%)
Hypercholesterolemia			58 (41.7%)
Smoking			68 (48.9%)
Family history of CAD			51 (36.6%)
History of MI			8 (5.7%)
History of revascularization			60 (43.1%)
Adenosine stress			17 (12.2%)

Data are shown as mean ± SD or number (%).

CAD: coronary artery disease, MI: myocardial infarction

**Table 2:** Clinical indications for myocardial perfusion imaging

	Inferior wall defect	Anterior wall defect
Diagnosis	41	8
Prognosis	24	4
Therapeutic control	45	3
Preoperative evaluation	10	4

### Scintigraphic imaging

Technetium-99m 1,2-bis [di-(2-ethoxyethyl) phosphino] ethane ([Tc-99m] tetrofosmin) one day stress-rest protocol was used. All patients had fasted for at least 4 hours and were previously advised to discontinue b-blockers, calcium-channel blockers, nitrates and avoid taking caffeine‑containing products for 24 hours before the radionuclide study. Exercise stress testing was preferred, using a modified Bruce protocol. In the presence of exercise limitations or contraindications, pharmacological stress with adenosine was used. Tc-99m tetrofosmin (370‑555 MBq) was administrated intravenously 1 min prior to peak exercise or 3 min into the adenosine infusion. Stress images were acquired first in the supine and second in the prone position, starting 15-30 min after exercise and 30-45 min after adenosine. A dual-headed, large-field-of view gamma camera (Philips, Forte Jetstream AZ) with a low-energy, high resolution collimator was used. The same acquisition settings and reconstruction parameters were used for both the supine and prone image acquisitions. In the presence of a disappearing defect in the prone position, rest imaging was omitted. Otherwise, 2 hours after the stress test, Tc-99m tetrofosmin (740-925 MBq) was infused intravenously and rest acquisition in the supine position was initiated 45‑60 min after the injection. Attenuation or scatter correction was not available and cine testing was not applied.

### Image analysis

The supine defects were classified as remaining or disappearing in the prone position. The wall defect improvement with positional change had to be complete to be considered as disappearing. New apparent anterior-anteroseptal defects in the prone position were attributed to sternal or rib attenuation artifact and did not alter the classification.

Processing and quantitative visual interpretation was performed using a 20-segment model^4,11^. Scintigrams were evaluated by observers with more than 15 years’ experience in nuclear cardiology. In case of difference in observers’ scores, there was agreement following discussion. The 5-point scoring system was used: 0 = normal; 1 = equivocal; 2 = moderate reduction of uptake; 3 = severe reduction of uptake; and 4 = no detectable tracer uptake. Based on the number and severity of segments with scores ≥ 2, the observers defined the study results as normal, probably normal, equivocal, probably abnormal or definitely abnormal^4,11^. To further define the results as normal or abnormal, the summed stress score (SSS) was calculated by adding the scores of the 20 segments of the stress Tc-99m tetrofosmin images^4,11^. SSS < 4 were considered normal, 4 to 8 mildly abnormal and >8 moderate to severely abnormal. The SSS had to be < 4 and the final scan interpretation had to be normal or probably normal, as any other case was considered abnormal^4^. Moreover, the SSS difference (SSS in supine image minus SSS in prone image) was calculated for each patient. Then the mean value of SSS difference for each defect group (disappearing or remaining defect group) was calculated. When rest imaging was done, segments were scored as well. Results were compared with clinical data and coronary angiography findings.

### Statistical analysis

The paired sample t-test was used to assess the statistical significance between the mean SSS derived from supine and prone studies. The mean SSS difference between the disappearing and remaining defect groups were compared using the independent samples t-test. The software program used for the statistical analysis was the SPSS statistics 19.0. A significance level of 0.01 was used.

The normality of the data was tested by using the Shapiro-Wilk test, which showed the data followed a normal distribution.

## Results

**Figure 1:**
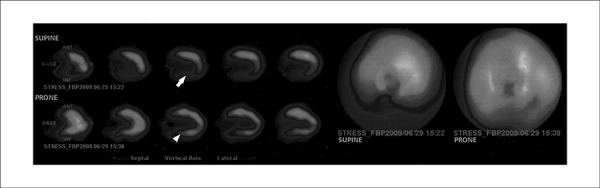
A 65-year old man with normal findings on coronary arteriography: an inferior wall defect in the supine position (arrow) that disappears with positional change (arrowhead), attributed to diaphragmatic attenuation artifact.

Inferior wall artifacts were seen in 114 male and in 6 female patients. In the present study, 94 of 120 patients with an inferior defect in the supine position (78.3%) showed normal prone interpretation (disappearing defect) and no further scintigraphy study was performed. The finding was attributed to diaphragmatic attenuation (Figure 1). Coronary angiography showed a patent right coronary artery (RCA) in 89 (74.2%) of 94 patients (true negative) and significant stenosis of the RCA in 5 (4.2%) of 94 patients (false negative). Among the study population, 26 of 120 patients (21.6%) showed abnormal prone interpretation (remaining defect) and the rest study was performed. In 19 (15.8%) of 26 patients, RCA stenosis was angiographically confirmed and the rest study disclosed evidence of transient ischemia or prior infarction (true positive). Of the remaining 7 patients (5.8%), the angiography showed normal RCA (false positive). The rest study demonstrated reversible defect in 4 of them, resulting in insufficient differentiation between ischemia and attenuation artifact. A fixed inferior wall defect was shown in the remaining 3 patients. Based on the patient’s clinical data and our experience, these findings were finally attributed to diaphragmatic attenuation. Among all 120 patients studied, an apparently new anterior-anteroseptal defect was observed in 8 patients (6.7%) in the prone position. This was attributed to sternal or rib attenuation, considering the angiographically confirmed normal patency of the corresponding coronary artery. Moreover, prone imaging tended to improve the specificity (92.7%) of detecting CAD in the inferior wall, without a significant reduction in sensitivity (79.2%).

Considering our data, 6 of 120 patients with an inferior wall defect in the supine position had prior infarction. Scintigraphy showed a “remaining” defect in the prone position in 4 of them (true positive). This finding was expected due to history of inferior wall infarction. In one female patient with prior inferior infarction, an unexpected prone normal interpretation was observed (false negative). Finally, one patient with anterior wall infarction showed perfusion defect improvement with positional change (true negative).

In patients with normal prone interpretation (disappearing defect) and angiographically confirmed patent RCA, the mean SSS of supine and prone studies were 9.35 ± 2.32 and 2.07 ± 1.28, respectively with the difference between them being statistically significant (p: 0.00). The mean SSS in patients with abnormal prone interpretation (remaining defect) and severely stenotic RCA were 11.74 ± 3.05 for the supine versus 10.95 ± 2.65 for the prone studies and the difference was marginally non-significant (p: 0.012). The statistical analysis is shown in Table 3. The mean SSS difference of the abnormal supine - normal prone and abnormal supine - abnormal prone scans was 7.28 ± 2.65 and 0.85 ± 1.19 respectively, with a significant difference (p: 0.00). Patients with inferior wall perfusion defect that had supine and prone acquisitions were more frequently males. Quantitative analysis did not change the scintigraphic results of visual interpretation and provided more accuracy.

**Table 3:** Data of the statistical analysis in the comparison of supine versus prone study

	Summed Stress Score		p Value
	Supine	Prone	
Disappearing defect by prone SPECT	9.35 ± 2.32	2.07 ± 1.28	0.00
Remaining defect by prone SPECT	11.77 ± 3.05	10.95 ± 2.65	0.012

**Figure 2:**
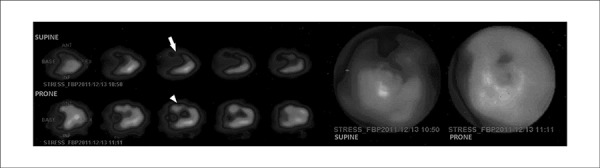
A 75-year old woman with no obstructive coronary artery disease: an anterior wall perfusion defect (arrow) that improves completely when changing from supine to prone (arrowhead). The defect was considered breast tissue attenuation artifact.

Performing the statistical analysis in women with an anterior wall defect was not feasible, due to the limited number of patients. Prone acquisition showed normal anterior wall activity in 12 of 19 patients (63.1%) and rest imaging was not performed. The finding was correctly attributed to breast attenuation (Figure 2). The normal patency of coronary vessels was angiographically confirmed. Two patients (10.5%) showed a defect that persisted despite positional change and the rest study was performed. Both had a history of anterior wall infarction. In spite of normal coronary angiograms and no history of CAD, 4 patients (21%) showed remaining defects (false positive). Moreover, one patient (5.2%) with total occlusion of the first diagonal branch showed prone normal tracer uptake (false negative).

Exercise on a treadmill was performed in 122 out of the 139 patients and achieved at least 85% of the maximum predicted heart rate (52 maximal and 70 sub-maximal stress tests). Pharmacological stress was performed with adenosine infusion in 17 patients. Positive exercise stress test suggestive of ischemia was observed in 29 patients. Scintigraphy showed reversible and fixed perfusion defects in 19 and in 2 patients respectively. In the remaining 8, normal perfusion imaging was detected. Fifteen patients with reversible perfusion defects had angiographically confirmed coronary artery stenosis, while 4 patients had a history of revascularization. Both cases with fixed defects had prior infarction, while angiography revealed borderline stenosis. Four patients with normal perfusion imaging had coronary artery stenosis, 3 had history of revascularization and one patient was highly suspected of cardiac syndrome X. Negative exercise stress test was observed in 93 patients. Myocardial imaging showed normal perfusion in 86 and abnormal in 7 cases. Observers diagnosed reversible and fixed defects in 3 and 4 cases respectively. One patient with reversible defect had angiographically confirmed stenosis, while the angiography showed normal vessel patency in the remaining 2; therefore, the finding was most likely attributed to attenuation artifact. Two patients with fixed defects had prior myocardial infarction, 1 had a history of revascularization and 1 was scheduled for angioplasty because of severe stenosis of the left anterior descending artery (LAD).

## Discussion

The present study confirms that prone imaging enhances the specificity and reduces artifact inferior wall abnormalities associated with supine-only study^3-5,9^, leading to more appropriate clinical decisions and shortening the hospital waiting period and patient discomfort^5,7,12^. Most importantly, rest myocardial perfusion study can be safely excluded in patients with an inferior wall “disappearing” defect by prone SPECT. This provides an excellent approach to limit radiation exposure by avoiding additional radiotracer infusion.

Soft tissue attenuation artifacts constitute a major shortcoming of myocardial perfusion imaging. Various techniques to improve specificity have been evaluated^1^, but to date there has been no clear definition of which is the best one^13,14^. It is generally accepted that attenuation artifacts are less frequent with Tc‑99m tracers than with thallium-201 (Tl-201)^15^. Prone imaging yields more accurate scintigraphic interpretations without any additional cost, it is inexpensive and it does not deliver any extra radiation to the patient^6^. It is associated with increased inferior and septal wall counts, less patient motion, patient discomfort and cardiac drift^12,16-18^. However, it is less suitable for females with large breasts and obese patients^2,19^. ECG-gating improves specificity of inferior wall disease detection and it additionally provides functional information^6,16^. The presence of normal wall motion in a fixed perfusion defect is usually consistent with attenuation artifact; however, small scars or nontransmural injuries may display this same imaging pattern^1^. Nevertheless, some authors believe that ECG-gating is the most practical method in routine investigations^20^. Direct attenuation correction systems are commercially available^1^. Although these systems tend to decrease the rate of equivocal interpretations to a greater extent than prone imaging, they require high-cost hardware and software products^1,5,6,21^.

The routine change of supine to prone imaging is a controversial matter, given the occasionally seen artifactual anterior‑anteroseptal wall prone defect^19,22,23^. This finding is presumably attributed to sternal and/or rib attenuation^1,4^ In the present study, this pitfall was observed in 8 out of the 120 patients (6.7%) The majority feels that prone should be considered only when imaging in the supine position raises the question of true inferior wall perfusion defect or artifact abnormality^4,19,22^.

The use of combined supine and prone quantitative imaging in overcoming diaphragmatic and/or breast attenuation artifacts has been evaluated before. Data from several researchers have shown significantly increased specificity without compromising sensitivity for the diagnosis of CAD^3,4,24^. This is in agreement with the results of our study, where a sensitivity of 79.2% and a specificity of 92.7% were shown. Katayama et al. have similarly demonstrated that prone stress Tl-201 study tends to improve the specificity of detecting coronary disease in the inferior wall. On the other hand, they showed that sensitivity is reduced when compared to stress-rest supine images^25^.

In our study population, rest acquisition was omitted in patients with defects on supine SPECT that disappear on prone imaging. However, few research groups performed stress and rest scans in all cases^4,9,10,18^^.^^19^. They all pointed out the excellent usefulness of combined supine and prone acquisitions on attenuation artifacts, which was also seen in our study. Segall and Davis have demonstrated that specificity for RCA was dramatically better (90% versus 66%) when patients were submitted to prone image acquisition compared to supine. Furthermore, the overall effect on the detection of CAD was an improved accuracy and higher specificity (82% *versus* 59%) without significant loss of sensitivity (75% *versus* 79%)^9^. In addition, Hayer et al concluded that patients with inferior wall defect in the supine position that was not present in the prone image had similar low risk of cardiac events, when compared with those that had normal supine only studies^4^. Recently Nishiyama et al, assessed the feasibility of combined imaging using a novel ultrafast cadmium zinc telluride (CZT) camera. They concluded that the combined supine and prone CZT SPECT yields significant gains in specificity and accuracy, whereas acquisition time is reduced by up to one fifth^26^.

False negative and false positive results of prone imaging were seen in 4.2% and 5.8% of our study population, respectively. The development of coronary collateral circulation could be a possible explanation for the false negative results. Thus, positional change may not always be sufficient to differentiate attenuation artifacts from CAD^6^.

Although some authors believe that prone imaging is associated with increased camera-to-chest wall distance and lower total myocardial counts when compared to supine position^2,19^, in this work prone image quality was very satisfactory. This is in agreement with a recent study by Gutstein et al. which showed that prone and supine imaging is associated with comparable good image quality in the non-obese population, even though half-time acquisition has been used^27^.

Anterior wall defects are most common in women. Although some believe that positional change mainly contributes to the disappearance rate of diaphragmatic attenuation^9,10^, it is a confirmed knowledge that combined supine and prone approach improve specificity and normalcy rates in women^24^. Although our study was limited to 19 patients only, 63.1% of the anterior wall defects disappeared in the prone image and subsequently, the rest perfusion study was properly obviated. Anterior wall defects in the supine acquisition that were absent with positional change tended to represent breast attenuation artifacts.

A number of strategies have been used to minimize dose in cardiac nuclear imaging. According to the “ALARA” philosophy, one should strive to keep radiation exposure As Low As Reasonably Achievable^28^. One enticing strategy is the use of Tc-99m agents and stress-first or stress-only protocols^28^. It seems that prone imaging provides an alternative imaging approach to reduce patient’s radiation exposure. Based on our study, prone acquisition correctly disclosed disappearing defects in 89 out of 120 patients with reduced uptake in the inferior wall and in 12 out of 19 women with reduced uptake in the anterior wall. The findings were considered to be diaphragmatic and breast tissue attenuation artifacts, respectively. Hence, prone SPECT imaging offers the possibility of avoiding the additional radiotracer infusion in an unnecessary rest study. This tends to reduce radiation dose by a factor of 4^29,30^, whilst providing similar prognostic information to normal rest-stress perfusion study^8,29^. Moreover, it saves time for both patients and busy departments, thus allowing additional nuclear medicine studies to be performed^29^.

Recently, a research group compared the inter-observer agreement between two experienced readers using supine alone *versus* combined supine/prone imaging. They showed improved inter-observer correlation and diagnostic agreement, by eliminating common artifacts, such as inferior wall attenuation, patient’s motion and interfering external activity. This will likely result in more uniform and standard care, which in addition to improvement in accuracy, will lead to fewer unnecessary additional tests^30^.

Ceylan Gunay et al have recently reported that an unnecessary rest Tc-99m methoxyisobutylisonitrile myocardial perfusion scintigraphy could be prevented in patients with complete disappearing inferior wall defect at stress prone imaging^7^. Similar to our results, they indicated that in patients with true defects, perfusion quantification was irrelevant to imaging position, as SSS of supine and prone stress studies were not different. This is of utmost importance, regarding the improvement of specificity, true positive rate and reliability of scintigraphic study.

Considering our data, there were 7 patients with an inferior wall and one patient with an anterior wall disappearing prone defect that underwent coronary angiography within a month after perfusion scintigraphy. They were highly suspected of having coronary artery stenosis because of their symptoms and risk factors. Angiograms showed no stenotic CAD. It seems that prone imaging might have an additional role in preventing unnecessary coronary angiograms and furthermore minimize radiation exposure, especially in low-risk patients. Recently, Worden et al. showed that patients with perfusion abnormalities during stress supine imaging that resolved during prone imaging are at low risk for cardiac death or myocardial infarction at medium-term follow up. Given that they seldom require invasive coronary angiography, broader application of prone imaging could lead to reduced exposure to the risks and expenses of unnecessary invasive procedures^31^.

### Limitations of the study

There are some limitations to the present study. The analysis is limited to the stress images of 120 patients only. Although rest imaging was performed in the presence of a remaining defect in the prone position and segments were scored as well, this was acquired only in the supine position. The study population was selected from a single center. Our results were related to supine and prone quantitative imaging without using gated assessment of wall motion or wall thickening. We investigated a mixed gender population regarding inferior wall perfusion defects, without performing any feasibility investigation. Although our data regarding female patients with an anterior wall defect are encouraging, the study sample is quite small and further trials are required on this issue. Here, we only present the preliminary results of an ongoing study.

## Conclusion

The addition of prone position to stress supine myocardial scintigraphy decreases the false positive rates and leads to more accurate results. Furthermore, it increases specificity without compromising sensitivity for the diagnosis of CAD. It has a key benefit of reducing the number of unnecessary rest studies performed, whilst minimizing radiation exposure, investigation time and costs. Moreover, it could possibly be a useful and practical method of obviating unnecessary referrals to coronary angiograms, especially in low-risk patients. There were no external funding sources for this study.
